# Supplementary material for: Digital Apps to Improve Mobility in Adults with Neurological Conditions: A Health App-Focused Systematic Review
Source: Healthcare (Basel). 2024 Apr 30;12(9):929. doi: 10.3390/healthcare12090929 (PMC11083333; doi:10.3390/healthcare12090929)
Supplement: Supplementary file 1 [file healthcare-12-00929-s001.zip › healthcare-2962058-supplementary.pdf]

*List of search terms*

| Search Category | Search Terms |
|---|---|
| Population | neurological |
| | neuro |
| | stroke |
| | brain injury |
| | TBI |
| | spinal cord injury |
| | SCI |
| | Parkinson's |
| | PD |
| | Multiple Sclerosis |
| | MS |
| Intervention | physiotherapy |
| | physio |
| | physiotherapist |
| | physical therapy |
| | rehabilitation |
| | rehab |
| | telehealth |
| | telerehabilitation |
| | task-specific |
| | task specific |
| | part practice |
| | re-training |
| | re training |
| | retraining |
| | mobility |
| | walking |
| | step |
| | stepping |
| | standing |
| | sit to stand |
| | balance |
| | coordination |
| | co-ordination |
| | co ordination |
| | dexterity |
| | wheelchair |
| | run |
| | running |
| | stair climbing |

**App Evaluation Framework**

Start of Block: App Classification

Q1.1 **App Classification**

Q1.2 App reference number

________________________________________________________________

Q1.3 Reviewer name

________________________________________________________________

Q1.4 Date reviewed

| | Month | Day | Year |
|---|---|---|---|
| Please Select: (1) | ▼ January (1 ... December (12) | ▼ 1 (1 ... 31 (31) | ▼ 1900 (1 ... 2049 (150) |

Q1.5 App name

________________________________________________________________

Q1.6 App store star rating

Select or type star rating (1) ☆ ☆ ☆ ☆ ☆

Q1.7 Number of ratings

________________________________________________________________

Q1.8 App developer

________________________________________________________________

Q1.9 App version

________________________________________________________________

Q1.10 Last update date (Android users):

| | Month | Day | Year |
| --- | --- | --- | --- |
| Select date: (1) | ▼ January (1 ... December (12) | ▼ 1 (1 ... 31 (31) | ▼ 1900 (1 ... 2049 (150) |

Q1.11 Last update date ($x$ months ago) (Apple users):

________________________________________________________________

Q1.12 Costs ($) (enter numerical value)

- ◯ Basic Version (1) ________________________________________________
- ◯ Upgrade Version (if relevant) (2) ________________________________________________
- ◯ Subscription (if relevant) (3) ________________________________________________

Q1.13 App store

- ◯ iTunes (1)
- ◯ Google Play (2)
- ◯ Other (3)

*Display This Question:*
*If App store = Other*

Q1.14 Please specify app store

________________________________________________________________

Q1.15 Platform app reviewed on

- ◯ iPhone (1)
- ◯ iPad (2)
- ◯ Android phone (3)
- ◯ Android tablet (4)

Q1.16 Requires companion device

- ○ No (1)
- ○ Yes (2)
- ○ Unsure (3)
- ○ Optional (4)

---

Q1.17 Affiliations

- ○ Unknown (1)
- ○ Commercial (2)
- ○ Government (3)
- ○ NGO (4)
- ○ University (5)

---

Q1.18 Brief Description

________________________________________________________________

________________________________________________________________

________________________________________________________________

________________________________________________________________

________________________________________________________________

---

End of Block: App Classification

---

Start of Block: Technical Aspects

Q2.1 **Technical Aspects**

---

Q2.2 Allows password protection

- ○ No (1)
- ○ Yes (2)
- ○ Unsure (3)

---

Q2.3 Requires login/account

- No (1)
- Yes (2)
- Unsure (3)

Q2.4 Needs web access to function

- No (1)
- Yes (2)
- Unsure (3)

Q2.5 Other technical considerations

________________________________________________________________

________________________________________________________________

________________________________________________________________

________________________________________________________________

________________________________________________________________

End of Block: Technical Aspects

Start of Block: Focus: Task-Specific Retraining

Q3.1 **Focus: Task-Specific Retraining**

Q3.2 **Does the app include exercises targeted at training**:

| | No (1) | Yes (2) |
| --- | --- | --- |
| **Sitting:** Includes part and whole task practice of sitting balance and endurance. This may include reaching in sitting, weight shifting in sitting, trunk movement in sitting (moving centre of mass over base of support in sitting) to challenge sitting balance. (1) | ○ | ○ |

| | No (1) | Yes (2) |
|---|---|---|
| **Standing Up:** Includes improving sit-to-stands via whole task or part task practice e.g. knees to stool, reaching in sitting, hips to table. Includes activities aimed at setting number of sit-to-stands as a goal e.g. number of sit to stands in a minute (2) | ○ | ○ |
| **Standing Balance:** Includes "static" standing practice such as parallel, tandem or single leg stance. Includes stepping outside of base of support or challenging base of support for balance goals e.g. stepping grid, step taps, standing on foam mat, reaching in standing, moving centre of mass over base of support in standing. Includes responding to changes in environment e.g. walking on uneven surfaces, responding to external perturbations, throwing/catching a ball, bouncing a ball. (3) | ○ | ○ |
| **Walking:** Includes part-practice stepping forwards, backwards and sideways. Includes other part practice such as stepping to a marker or over an obstacle, step ups. weaving around obstacles, dual tasking whilst walking, changing directions, etc. Includes treadmill training and overground walking to support functional walking goal. (4) | ○ | ○ |
| **Stair Climbing:** Includes whole and part-task stair climbing practice e.g. step taps for goal of stair mobility, step ups, rapidly descending stairs. (5) | ○ | ○ |
| **Running:** Includes pre-running and other high level tasks such as jumping, hopping and agility training. Includes running whole and part-task practice. (6) | ○ | ○ |

| | No (1) | Yes (2) |
|---|---|---|
| **Strength:** Includes strength training exercises. This includes single joint or non-functional position strengthening exercises. Include use of weights, theraband slidesheets, skateboard, etc. (7) | ○ | ○ |
| **Other:** Does the app target any other aspects of task-specific retraining (8) | ○ | ○ |

---

*Display This Question:*
*If Does the app include exercises targeted at training: = <strong>Other:</strong> Does the app target any other aspects of task-specific retraining [ Yes ]*

Q3.3 Please comment what other aspects of task-specific training the app targets

________________________________________________________________

________________________________________________________________

________________________________________________________________

________________________________________________________________

Q3.4 Does the app include photos or videos of exercises?

○ No (1)

○ Yes (2)

---

*Display This Question:*
*If Does the app include photos or videos of exercises = [ Yes ]*

Q3.5 Are there patients in these photos/videos?

○ No (1)

○ Yes (2)

---

**End of Block: Focus: Task-Specific Retraining**

---

**Start of Block: Telehealth Features**

Q4.1 **Telehealth Features**

---

Q4.2 **Education:**
Is the app designed to provide education on:
- A condition or diagnosis?

- The benefits of exercise/physical activity
- The evidence behind the exercises prescribed.

○ No (1)

○ Yes (2)

○ Links to more information (3)

Q4.3 **Assessment:**
Is the app designed to assist with completing and tracking objective assessments such as completing a berg balance test or six minute walk test?

○ No (1)

○ Yes (2)

Q4.4 **Customisation:**
Does the app allow for customisation of exercise prescription?

○ **Pre-set program (no customization):** Option/s for pre-determined program only. Nil customisation. (1)

○ **Pre-set program with modifiable dose OR difficulty:** Option/s for pre-determined program where dosage (sets/reps/time) can be customised OR where exercise difficulty can be modified e.g. increasing weight, reducing support (2)

○ **Partially customisable:** Customisable exercises without the option to customize dose or difficulty (3)

○ **Completely Customisable:** Prescriber able to select from a range of exercises to create a program, including type, dosage and difficulty. (4)

○ **Other:** Falls between categories (5)

*Display This Question:*
*If Customisation: Does the app allow for customisation of exercise prescription? = <strong>Other:</strong> Falls between categories*

Q4.5 Please describe other rehabilitation features if app falls between categories

________________________________________________________________
________________________________________________________________
________________________________________________________________
________________________________________________________________
________________________________________________________________

Q4.6 **Monitoring**

| | No (1) | Yes (2) |
|---|---|---|
| **User monitoring:** Is the app designed for users to keep a record of their own behaviour by providing statistical data or recordings of activity within the app itself? (1) | ○ | ○ |
| **Therapist/ prescriber remotely monitoring (following data sharing):** Is the app designed to allow the user to share data directly with the prescriber? (2) | ○ | ○ |
| **Therapist/ prescriber remotely monitoring (without data sharing):** Is the app designed to allow the therapist/ prescriber to view data directly and without the user manually sharing? (3) | ○ | ○ |
| **Therapist/ prescriber remotely providing feedback:** Does the app allow for the prescriber/therapist to provide feedback or comments to the user remotely? (4) | ○ | ○ |
| **Therapist/ prescriber remotely altering program** Does the app allow for the therapist/ prescriber to remotely alter the program? i.e. to progress/regress exercises or dosage. (5) | ○ | ○ |

*Display This Question:*

*If Therapist/ prescriber remotely monitoring (following data sharing): Is the app designed to allow the user to share data directly with the prescriber? = Yes [2]*

Q 4.6.2.1 Is there a requirement for the user to provide consent for data sharing with prescriber?

○ No (1)

○ Yes (2)

○ N/A (3)

Q4.7 **Goal Setting:**
Does the app allow for goal setting of SMART (Specific, Measurable, Achievable, Realistic, Time-bound) goals that are individualised and person-centered with the user?

- No (1)
- Yes (2)

Q4.8 **Number of exercises offered:**
Note: Exercises should be different and not progressions/regressions of the same exercise. Task-specific exercises must work towards improving a particular functional task i.e. sitting, standing, balancing, walking, stair climbing, running.

| | |
|---|---|
| How many exercises does the app offer? (1) | 1-5 exercises (1)<br>6-10 exercises (2)<br>11-20 exercises (3)<br>>20 exercises (4) |
| How many task-specific exercises does the app offer? (2) | 1-5 exercises (1)<br>6-10 exercises (2)<br>11-20 exercises (3)<br>>20 exercises (4) |
| How many of these task-specific exercises are you likely to prescribe for this population? (3) | 1-5 exercises (1)<br>6-10 exercises (2)<br>11-20 exercises (3)<br>>20 exercises (4) |

Q4.9 Comment on any other significant telehealth features that the app offers

________________________________
________________________________
________________________________
________________________________
________________________________

End of Block: Telehealth Features

**Start of Block: Impairment classification**

Q5.1 **Impairment Classification**

Q5.2 Is the app targeted towards any of the following groups (select multiple)

☐ **General rehabilitation:** Exercises / app not targeted at a specific group. Exercises are general and can be used by anyone. (1)

☐ **Neurological rehabilitation:** Exercises / app targeted at a neurological population. Not specific to a certain condition. (2)

☐ **Diagnostic group / health condition:** Exercises / app clearly targeted at a specific population group through information, affiliations, etc for example Parkinson's Disease, MS, Stroke, etc. (3)

☐ **Older people:** Exercises / app clearly targeted at older/ elderly/ frail population through images, information, affiliations, etc. (4)

☐ **None of the above / unclear** (5)

---

*Display This Question:*

*If Is the app targeted towards any of the following groups (select multiple) = <strong>None of the above / unclear</strong>*

Q5.3 Please specify the specific diagnostic group / health condition that the app is targeted at or how it is unclear

________________________________________________________________

---

*Display This Question:*

*If Is the app targeted towards any of the following groups (select multiple) = <strong>None of the above / unclear</strong>*

Q5.4 Please specify why the app is not targeted at a specific group. (This app may need to be further reviewed to establish if it meets the inclusion criteria).

________________________________________________________________

________________________________________________________________

________________________________________________________________

________________________________________________________________

________________________________________________________________

---

Q5.5 **Impairment classification**

| | No (1) | Maybe (2) | Yes (3) |
|---|---|---|---|
| **Are the exercises within the app suitable for people with a moderate-severe physical impairment?** Physical impairment includes: weakness, coordination, reduced range of motion/contracture, sensory impairments. *Examples include:* - Exercises in a gravity eliminated plane - Exercises utilising low friction equipment such as slide sheets and mini skateboard - Instructions on safe set up of exercises and utilising a carer for assistance. (1) | ○ | ○ | ○ |
| **Is the app designed for use by people with a moderate-severe physical impairment?** Physical impairment includes: weakness, coordination, reduced range of motion/contracture, sensory impairments. *Examples include:* - Large buttons on display - Minimal tapping of buttons to navigate/use app - Option for use of a switch device to navigate app - Option for voice-navigation of app. (2) | ○ | ○ | ○ |

| | No (1) | Maybe (2) | Yes (3) |
|---|---|---|---|
| **Is the app designed for use by people with a moderate-severe language impairment?** Language impairment includes receptive and expressive impairments. *Examples include:* - - Option for voice over to read options on screen. - - Option for voice commands to navigate screen. - Simple language used in app. No large block writing. - Images, pictures and/or videos utilised to simplify information. (3) | ○ | ○ | ○ |
| **Is the app designed for use by people with a moderate-severe perceptual and/or visual impairment?** *Examples include:* - - Colour inversion. - Options for larger text or zooming in. - Option for voice over to read options on screen. (4) | ○ | ○ | ○ |

| | No (1) | Maybe (2) | Yes (3) |
|---|---|---|---|
| **Is the app designed for use by people with a moderate-severe cognitive impairment?** Cognitive impairments may include reduced processing speed, impaired short term memory, inattention (including neglect) and problem solving impairments. *Examples include:* - Simple design, not many buttons/options, intuitive to use. - Includes reminders to complete activity, share data and acknowledge achievements. - Tracks activity/number of repetitions. - Information displayed in an easy to understand way through images/videos. (5) | o | o | o |
| **Is the app designed for use by people with a moderate-severe behavioural impairment?** Behavioural impairment may include distractibility, reduced ability to regulate emotions, reduced insight. *Examples include:* - Reminders during exercise to keep person on task - Recognition of achievements. - Reminders to complete exercise - Comparison with others with a similar injury - Difficulty levels within the app. (6) | o | o | o |

Q5.6 Comment on any other ways the app accommodates for different levels of physical, cognitive or sensory impairment.

________________________________________________________________

________________________________________________________________

________________________________________________________________

________________________________________________________________

________________________________________________________________

End of Block: Impairment classification

Start of Block: MARS: Section A: Engagement

Q6.1 **MARS: Section A: Engagement**

Q6.2 **Entertainment:**
Is the app fun/entertaining to use? Does it use any strategies to increase engagement through entertainment (e.g. through gamification)?
Other examples to increase engagement include through providing fun games, videos or activities.

○ 1 = Dull, not fun or entertaining at all (1)

○ 2 = Mostly boring (2)

○ 3 = OK, fun enough to entertain use for a brief time (< 5 minutes) (3)

○ 4 = Moderately fun and entertaining, would entertain user for some time (5-10 minutes) (4)

○ 5 = Highly entertaining and fun, would stimulate repeat use (5)

Q6.3 **Interest:** Is the app interesting to use? Does it use any strategies to increase engagement by presenting its content in an interesting way? It is important to distinguish between fun and interesting in this context. An example is using animations instead of plain text.

○ 1 = Not interesting at all (1)

○ 2 = Mostly uninteresting (2)

○ 3 = OK, neither interesting nor uninteresting; would engage user for a brief time ( (3)

○ 4 = Moderately interesting; would engage user for some time (5-10 minutes) (4)

○ 5 = Very interesting, would engage user in repeat use (5)

Q6.4 **Customisation:**
Does it provide/retain all necessary settings/preferences for app features (e.g. sound, content, notifications, etc.)?
Does the app allow for tailoring/modification of setting according to individual characteristics or preferences for features.
To justify a rating of 4 or 5 the app would include customisation options that are not essential to the use of the app but are nice additions.

- ◯ 1 = Does not allow any customisation or requires setting to be input every time (1)
- ◯ 2 = Allows insufficient customisation limiting functions (2)
- ◯ 3 = Allows basic customisation to function adequately (3)
- ◯ 4 = Allows numerous options for customisation (4)
- ◯ 5 = Allows complete tailoring to the individual's characteristics/preferences, retains all settings (5)

---

Q6.5 **Interactivity:**
Does it allow user input, provide feedback, contain prompts (reminders, sharing options, notifications, etc.)?
Note: these functions need to be customisable and not overwhelming in order to be perfect.
It is important to distinguish between customisation (settings) and interactivity (features).
Examples include: in-app community, sharing with other users.

- ◯ 1 = No interactive features and/or no response to user interaction (1)
- ◯ 2 = Insufficient interactivity, or feedback, or user input options, limiting functions (2)
- ◯ 3 = Basic interactive features to function adequately (3)
- ◯ 4 = Offers a variety of interactive features/feedback/user input options (4)
- ◯ 5 = Very high level of responsiveness through interactive features/feedback/user input options (5)

---

Q6.6 **Target Group:**
Is the app content (visual information, language, design) appropriate for your target audience?
*Note:* For the purposes of this question, the target group is healthy adults.

- ◯ 1 = Completely inappropriate/unclear/confusing (1)
- ◯ 2 = Mostly inappropriate/unclear/confusing (2)
- ◯ 3 = Acceptable but not targeted. May be inappropriate/unclear/confusing (3)
- ◯ 4 = Well-targeted, with negligible issues (4)
- ◯ 5 = Perfectly targeted, no issues found (5)

---

**End of Block: MARS: Section A: Engagement**

---

**Start of Block: MARS: Section B: Functionality**

Q7.1 **MARS: Section B: Functionality**

Q7.2 **Performance:**
How accurately/fast do the app features (functions) and components (buttons/menus) work?
Looking for prompt, timely responses for different features and buttons.
In order to rate this, you need to physically touch every button on the app and go through all the features.

- ○ 1 = App is broken; no/insufficient/inaccurate response (e.g. crashes/bugs/broken features, etc.) (1)
- ○ 2 = Some functions work, but lagging or contains major technical problems (2)
- ○ 3 = App works overall. Some technical problems need fixing/Slow at times (3)
- ○ 4 = Mostly functional with minor/negligible problems (4)
- ○ 5 = Perfect/timely response; no technical bugs found/contains a 'loading time left' indicator (5)

---

Q7.3 **Ease of use:**
How easy is it to learn how to use the app; how clear are the menu labels/icons and instructions?

- ○ 1 = No/limited instructions; menu labels/icons are confusing; complicated (the app is practically impossible to work out how to use the app) (1)
- ○ 2 = Useable after a lot of time/effort (the app is quite difficult to figure out, whether or not it has a tutorial) (2)
- ○ 3 = Useable after some time/effort(the app is relatively easy to learn how to use but it does take a little bit of time) (3)
- ○ 4 = Easy to learn how to use the app (or has clear instructions) (if the app is not perfectly intuitive, it has a solid tutorial) (4)
- ○ 5 = Able to use app immediately; intuitive; simple (the app is perfectly intuitive) (5)

---

Q7.4 **Navigation:**
Is moving between screens logical/accurate/appropriate/uninterrupted; are all necessary screen links present?
Examples of apps with poor navigation include missing buttons, missing links between screens, log in information but not registration information.
Consider how difficult it is to move from one screen e.g. inbox back to your initial screen.

- ○ 1 = Different sections within the app seem logically disconnected and random/confusing/navigation is difficult (1)
- ○ 2 = Usable after a lot of time/effort (2)
- ○ 3 = Usable after some time/effort (3)
- ○ 4 = Easy to use or missing a negligible link (4)
- ○ 5 = Perfectly logical, easy, clear and intuitive screen flow throughout, or offers shortcuts (5)

---

Q7.5 **Gestural design:**
Are interactions (taps/swipes/pinches/scrolls) consistent and intuitive across all components/screens?
Double tap, pinches and swipes are popular but e.g. triple taps might not make much sense.
If there are any gestures that are not widely used and are not intuitive e.g. tap and hold, they should be discussed in the tutorial and the app would still not get a rating of 5, it would get a 4 at most.

- ○ 1 = Completely inconsistent/confusing (1)
- ○ 2 = Often inconsistent/confusing (2)
- ○ 3 = OK with some inconsistencies/confusing elements (3)
- ○ 4 = Mostly consistent/intuitive with negligible problems (4)
- ○ 5 = Perfectly consistent and intuitive (5)

---

**End of Block: MARS: Section B: Functionality**

---

**Start of Block: MARS: Section C: Aesthetics**

Q8.1 **MARS: Section C: Aesthetics**

---

Q8.2 **Layout:**
Is arrangement and size of buttons/icons/menus/content on the screen appropriate or zoomable if needed?
Looks at the menu arrangement of each screen on the app, whether or not the buttons are accessible, easy to touch and don't create clutter by being too big.

- ○ 1 = Very bad design, cluttered, some options impossible to select/locate/see/read device display not optimised (1)
- ○ 2 = Bad design, random, unclear, some options difficult to select/locate/see/read (2)
- ○ 3 = Satisfactory, few problems with selecting/locating/seeing/reading items or with minor screen size problems (3)
- ○ 4 = Mostly clear, able to select/locate/see/read items (4)
- ○ 5 = Professional, simple, clear, orderly, logically organised, device display optimised. Every design component has a purpose (5)

---

Q8.3 **Graphics:**
How High is the quality/resolution of graphics used for buttons/icons/menus/content?
Mainly looking at the quality of the resolution and the professionalism of the app design. The highest quality graphics often leave us with a sense of innovation.

- ○ 1 = Graphics appear amateur, very poor visual design - disproportionate, completely stylistically inconsistent (1)
- ○ 2 = Low quality/low resolution graphics; low quality visual design - disproportionate, stylistically inconsistent (2)
- ○ 3 = Moderate quality graphics and visual design (generally inconsistent in style) (3)
- ○ 4 = Mostly clear, able to select/locate/see/read items (4)
- ○ 5 = Professional, simple, clear, orderly, logically organised, device display optimised. Every design component has a purpose. (5)

---

Q8.4 **Visual appeal:**
How good does the app look?
Mostly looking at the colour choice of the app, whether or not the colour scheme is appealing and the overall sense that the app leaves the user with, if the app is memorable and stands out amongst others.

- ○ 1 = No visual appeal, unpleasant to look at, poorly designed, clashing/mismatched colours (1)
- ○ 2 = Little visual appeal – poorly designed, bad use of colour, visually boring (2)
- ○ 3 = Some visual appeal – average, neither pleasant, nor unpleasant (3)
- ○ 4 = High level of visual appeal – seamless graphics – consistent and professionally designed (4)
- ○ 5 = As above + very attractive, memorable, stands out; use of colour enhances app features/menus (5)

---

**End of Block: MARS: Section C: Aesthetics**

---

**Start of Block: MARS: Section D: Information**

Q9.1 **MARS: Section D: Information**

---

Q9.2 **Accuracy of app description (in app store):**
Does app contain what is described?
Check the description on the app store.
Often these are misleading and can make promises the app is not able to achieve.
An excellent app description should contain quality information about the app, honestly listing and explaining its components as well as who developed it and where/how users can reach out if they have any problems.

- ○ 1 = Misleading. App does not contain the described components/functions or has no description (1)
- ○ 2 = Inaccurate. App contains very few of the described components/functions (2)
- ○ 3 = OK. App contains some of the described components/functions (3)
- ○ 4 = Accurate. App contains most of the described components/functions (4)
- ○ 5 = Highly accurate description of the app components/functions (5)

---

Q9.3 **Goals:**
Does app have specific, measurable and achievable goals (specified in app store description or within the app itself)?
If goals are measurable, there should be some way of tracking progress towards goal achievement (rating of 4 or 5).
Not every app states the goals explicitly but it should be obvious in the app description or in the app itself when you first enter.

- ○ 1 = App has no chance of achieving its stated goals (1)
- ○ 2 = Description lists some goals, but app has very little chance of achieving them (2)
- ○ 3 = OK. App has clear goals, which may be achievable (3)
- ○ 4 = App has clearly specified goals, which are measurable and achievable (4)
- ○ 5 = App has specific and measurable goals, which are highly likely to be achieved (5)
- ○ N/A = Description does not list goals, or app goals are irrelevant to research goal (e.g. using a game for educational purposes) (6)

---

Q9.4 **Quality of information:**
Is app content correct, well written, and relevant to the goal/topic of the app?
Important to identify this in order to avoid potential harm to the user.

- ○ 1 = Irrelevant/inappropriate/incoherent/incorrect (1)
- ○ 2 = Poor. Barely relevant/appropriate/coherent/may be incorrect (2)
- ○ 3 = Moderately relevant/appropriate/coherent/and appears correct (3)
- ○ 4 = Relevant/appropriate/coherent/correct (4)
- ○ 5 = Highly relevant, appropriate, coherent, and correct (5)
- ○ N/A = There is no information within the app (6)

---

Q9.5 **Quantity of information:**
Is the extent coverage within the scope of the app; and comprehensive but concise?
How much is in there? Apps should be brief, concise and clear. Highly rated apps may have expandable information – it is an option for not overwhelming people with information

- 1 = Minimal or overwhelming (1)
- 2 = Insufficient or possibly overwhelming (2)
- 3 = OK but not comprehensive or concise (3)
- 4 = Offers a broad range of information, has some gaps or unnecessary detail; or has no links to more information and resources (4)
- 5 = Comprehensive and concise; contains links to more information and resource (5)
- N/A = There is no information within the app (6)

---

Q9.6 **Visual information:**
Is visual explanation of concepts – through charts/graphs/images/videos, etc. – clear, logical, correct?
Whilst sometimes apps use graphs, pie charts, tables, etc. to present information, sometimes they can be confusing to interpret/read.
Sometimes an explanation to the user is needed about what the levels mean, the colours, etc.
A video of a lecture is not included in this as this domain is targeting visual representation of written text (or information that could be written text).
Does it communicate information visually in an interactive and interesting way. That information needs to be accurate and get the message across.

- 1 = Completely unclear/confusing/wrong or necessary but missing (1)
- 2 = Mostly unclear/confusing/wrong (2)
- 3 = OK but often unclear/confusing/wrong (3)
- 4 = Mostly clear/logical/correct with negligible issues (4)
- 5 = Perfectly clear/logical/correct (5)
- N/A = There is no visual information within the app (e.g. it only contains audio, or text) (6)

---

Q9.7 **Credibility:**
Does the app come from a legitimate source (specified in app store description or within the app itself)?
Look at who developed the app and whether or not they are an expert in the field and have the authority to provide the information.
If it was developed by a small NGO/institution or specialised commercial business it would score a 3 but if they provided references to sources from university or government bodies, they could score higher according to the scale.

- ○ 1 = Source identified but legitimacy/trustworthiness of source is questionable (e.g. commercial business with vested interest) (1)
- ○ 2 = Appears to come from a legitimate source, but it cannot be verified (e.g. has no web page) (2)
- ○ 3 = Developed by small NGO/institution (hospital/centre, etc.) /specialised commercial business, funding body (3)
- ○ 4 = Developed by government, university or as above but larger in scale (4)
- ○ 5 = Developed using nationally competitive government or research funding (e.g. Australian Research Council, NHMRC) (5)

---

Q9.8 **Evidence base:**
Has the app been trialled/tested; must be verified by evidence (in published scientific literature)?
Need to do homework – look on google scholar or a research database and search the app's name to identify if there is any published research on the app.

- ○ 1 = The evidence suggests the app does not work (1)
- ○ 2 = App has been trialled (e.g., acceptability, usability, satisfaction ratings) and has partially positive outcomes in studies that are not randomised controlled trials (RCTs), or there is little or no contradictory evidence (2)
- ○ 3 = App has been trialled (e.g., acceptability, usability, satisfaction ratings) and has positive outcomes in studies that are not RCTs, and there is no contradictory evidence (3)
- ○ 4 = App has been trialled and outcome tested in 1-2 RCTs indicating positive results (4)
- ○ 5 = App has been trialled and outcome tested in > 3 high quality RCTs indicating positive results (5)
- ○ N/A = The app has not been trialled/tested (6)

---

**End of Block: MARS: Section D: Information**

---

**Start of Block: MARS: Section E: Subjective Quality**

Q10.1 **MARS: Section E: Subjective Quality**

---

Q10.2 **Would you recommend this app to people who might benefit from it?**
Consider the target population (adults with a neurological condition).

- 1 = Not at all I would not recommend this app to anyone (1)
- 2 = There are very few people I would recommend this app to (2)
- 3 = Maybe. There are several people whom I would recommend it to (3)
- 4 = There are many people I would recommend this app to (4)
- 5 = Definitely I would recommend this app to everyone (5)

---

Q10.3 **How many times do you think you would use this app in the next 12 months if it was relevant to you?**
Consider the number of times you would use an app before it becomes repetitive and then deleted (if it was relevant to you).

- None (1)
- 1-2 (2)
- 3-10 (3)
- 10-50 (4)
- >50 (5)

---

Q10.4 **Would you pay for this app?**
It is not a question around whether the price of the app seems reasonable but if the quality of the app is worth paying for.

- Yes (1)
- Maybe (2)
- No (3)

---

Q10.5 **What is your overall star rating of the app?**
1 = One of the worst apps I've used
3 = Average
5 = One of the best apps I've used
Select (1) ☆ ☆ ☆ ☆ ☆

---

End of Block: MARS: Section E: Subjective Quality

Start of Block: MARS: Section F: App-Specific

Q11.1 **MARS: Section F: App-Specific**

---

Q11.2 **App-Specific:**

| | No (1) | Unsure (2) | Yes (3) |
|---|---|---|---|
| **Awareness:** This app is likely to increase awareness of the importance of addressing: - Physical function - Inactivity (1) | ○ | ○ | ○ |
| **Knowledge:** This app is likely to increase knowledge/understanding of: - Task-Specific retraining exercises - Repetitive practice - The benefits of exercise (2) | ○ | ○ | ○ |
| **Attitudes:** This app is likely to change attitudes toward improving: - Physical Function - Time being Physically Active (3) | ○ | ○ | ○ |
| **Intention to change:** This app is likely to increase intentions/motivation to: - Increase number of repetitions of practice - Increase active time (4) | ○ | ○ | ○ |
| **Help seeking:** Use of this app is likely to encourage further help seeking for: - Improving physical function - Staying physically active (5) | ○ | ○ | ○ |
| **Behaviour change:** Use of this app is likely increase: - Home practice of task-specific exercises - Physical activity (6) | ○ | ○ | ○ |

End of Block: MARS: Section F: App-Specific

Start of Block: ABACUS: Section 1: Knowledge & Information

Q12.1 **ABACUS: Section 1: Knowledge & Information**

Q12.2 **Knowledge and Information**

| | No (1) | Yes (2) |
|---|---|---|
| **Does the app have the ability to customize and personalize some features?** Elements of the app can be personalised through specific tools or functions that are specific to the individual using the app. *Examples include:* - To select a disease type from among several available and the to follow a specific path or set of tools or systems. - To select to receive emails or texts of a specific nature. - To choose "yes" or "no" to a specific capability of the app - To create a personalized exercise plan. (1) | ○ | ○ |
| **Was the app created with expertise and/or does the app provide information that is consistent with national guidelines?** This would be found in the about section or generally in the app. *Examples include:* - Does the app suggest a high number of repetitions of practice? - Does the app suggest not using upper limb support when training balance? - Is there any evidence that the app was created by an expert? (physio/professional body/university) (2) | ○ | ○ |
| **Does the app ask for baseline information?** This includes BMI, weight, exercise, age, condition, level of function - This might be at the set-up phase or in a profile setting. (3) | ○ | ○ |

| | No (1) | Yes (2) |
|---|---|---|
| **Does the app provide instruction on how to perform the behaviour?** The app is clear in telling the person to perform a behaviour or predatory behaviours, either verbally, through video, or in written form. NB: the behaviour that is seeking to be changed, not information on how to use the app. *Examples include:* - Showing person how to use equipment, how to set up for safe exercise, instructions on suitable clothing and general tips. (4) | ○ | ○ |
| **Does the app provide information about the consequences of continuing and/or discontinuing behaviour?**The app gives user information about the consequences of behaviour in general, this includes information about the relationship between the behaviour and its possible or likely consequences in the general case. This information can be general or personalised.- Consequences may include health, feelings, functional consequences etc. (5) | ○ | ○ |

End of Block: ABACUS: Section 1: Knowledge & Information

Start of Block: ABACUS: Section 2: Goals & Planning

Q13.1 **ABACUS: Section 2: Goals & Planning**

Q13.2 **Goals and Planning**

| | No (1) | Yes (2) |
|---|---|---|
| **Does the app ask for willingness for behaviour change?** Is there a feature during setup where you describe how ready you are for behaviour change? *Examples include:* - This may be in the form of a scale of readiness or in a question that asks the user to describe how ready they are. (1) | ○ | ○ |
| **Does the app allow for setting up of goals?** The person is encouraged to make a behavioural resolution. The person is encouraged to set a general goal that can be achieved by behavioural means. This includes sub-goals or preparatory behaviours and/or specific contexts in which the behaviour will be performed. The behaviour in this technique will be directly related to or be a necessary condition for the target behaviour. - This is the explicit noting of a goal or choosing a goal from one provided within the app. - Goals should ideally be SMART in nature. (2) | ○ | ○ |
| **Does the app have the ability to review goals, update and change when necessary?** Involves a review or analysis of the extent to which previously set behavioural goals (regardless of short or long) were achieved. - This is where a goal can be changed. This allows people to act on previously set goals and then revise or adjust where needed. (3) | ○ | ○ |

End of Block: ABACUS: Section 2: Goals & Planning

**Start of Block: ABACUS: Section 3: Feedback & Monitoring**

Q14.1 **ABACUS: Section 3: Feedback & Monitoring**

Q14.2 **Feedback and Monitoring**

| | No (1) | Yes (2) |
|---|---|---|
| **Does the app give the user the ability to quickly and easily understand the difference between current action and future plans?** Allows user to see how they are tracking against a goal and to see the difference between what they want to do and what they are currently doing. This will give some feedback on where they are at and what they need to change to get to where they want to be. - This could be in the form of a graph or some other visual describing how close the user is to meeting their goals. (1) | ○ | ○ |
| **Does the app have the ability to allow the user to easily self-monitor behaviour?** The app allows for a regular monitoring of the activity. This is done by keeping a record or log of activity that can be referred back to at a later date. *Examples include:* - Connects with watch that records daily steps that can be reviewed. - Allows for easy logging of exercise (2) | ○ | ○ |
| **Does the app have the ability to share behaviours with others (including social media or forums) and/or allow for social comparison?** The app allows the person to share his or her behaviours on social media or in forums. This could also include a buddy system or a leader board. *Examples include:* - Share with Facebook or other socials. - Tell the user that they are doing x and at this time, other people like them are doing y. (3) | ○ | ○ |

| | No (1) | Yes (2) |
|---|---|---|
| **Does the app have the ability to give the user feedback?** The app is able to provide the person with feedback, comments, or data about their own recorded behaviour. This might be automatic or could be personal. - Does the app automatically provide feedback when a person reaches a nominated goal e.g. number of steps per day, number of reps achieved. (4) | ○ | ○ |
| **Does the app have the ability to export data from app?** The app allows for the export of information and progress to an external user. *Examples include:* - Export to a computer or to another user such as a physiotherapist - Sharing to Facebook does not count. (5) | ○ | ○ |
| **Does the app provide a material or social reward or incentive?** App provides rewards for attempts at achieving a behavioural goal. This might include efforts made toward achieving the behaviour or progress made in preparatory steps toward the behaviour or in achieving a goal. *Examples include:* - Social or public, for example, congratulating the person for each day that they meet their exercise target. (6) | ○ | ○ |
| **Does the app provide general encouragement?** The app provides general encouragement and positive reinforcement on actions leading to the goal. *Examples include:* - Achievement badges - Telling the user that they are a certain percentage closer to their goal. (7) | ○ | ○ |

End of Block: ABACUS: Section 3: Feedback & Monitoring

Start of Block: ABACUS: Section 4: Actions

Q15.1 **ABACUS: Section 4: Actions**

Q15.2 **Actions**

| | No (1) | Yes (2) |
|---|---|---|
| **Does the app have reminders and/or prompts or cues for activity?** The app prompts the user to engage in the activity. The app has the ability to give notifications or reminders to cue the behaviour. *Examples include:* - A watch reminding you to stand. - An app telling you it is time to exercise. (1) | ○ | ○ |
| **Does the app encourage positive habit formation?** The app prompts explicit rehearsal and repetition of the behaviour– not just tracking or logging. - An example of this are the couch to 5 km apps that provide a training schedule (2) | ○ | ○ |
| **Does the app allow or encourage for practice or rehearsal, in addition to daily activities?** App does not have a lock on activities or a number that you cannot exceed daily. - This would include allowing the user to undertake extra activities in a single day. (3) | ○ | ○ |
| **Does the app provide opportunity to plan for barriers?** The app encourages the person to think about potential barriers and identify ways of overcoming them. - Exercise app might promote positive sleep behaviours to have enough energy to exercise the next day. (4) | ○ | ○ |

| | No (1) | Yes (2) |
|---|---|---|
| **Does the app assist with or suggest restructuring the physical or social environment?** The app prompts the person to alter the environment in ways so that it is more supportive of the target behaviour. *Examples include:* - App suggests taking their running shoes to work. - App suggests maintaining safe home set up for task-specific practice (i.e. not packing away the set up, having a clear defined exercise space). (5) | ○ | ○ |
| **Does the app assist with distraction or avoidance?** The app gives suggestions and advice on how the person can avoid situations or distract themselves when trying to reach their goal. - Exercise app may suggest exercising before having morning coffee or watching TV. (6) | ○ | ○ |

End of Block: ABACUS: Section 4: Actions

Start of Block: Other Comments

Q16.1 **Optional: Other Comments**

Q16.2 **Optional: Describe any other positive comments you have about the app**
*Examples include:*
- Lite version just as good as pro or paid version.
- Wheelchair exercises
- Offered in other languages

________________________________________________________________
________________________________________________________________
________________________________________________________________
________________________________________________________________
________________________________________________________________

Q16.3
**Optional: Describe any other neutral comments you have about the app**
*Examples include:*
- Elaborating on things you are unsure about

________________________________________________________________

Q16.4 **Optional: Describe any negative comments you have about the app**
*Examples include:*
- Constant advertising
- Chat room topics not relevant
- Not targeted at Australian market

**End of Block: Other Comments**

*Aps downloaded for screening and exclusion reasons*

| Code | Primary Reason for exclusion | Number of Apps |
|---|---|---|
| 1 | Not targeted at population group | 54 |
| 2 | No/limited lower limb exercises in app | 17 |
| 3 | No follow up from developer | 13 |
| 4 | For clients attending a particular practice/location only | 6 |
| 5 | App not opening/crashing | 7 |
| 6 | App no longer available on app store | 9 |
| 7 | Full app features not available in Australia | 5 |
| 8 | Not in English | 6 |
| 9 | Requires a companion device not accessible to the research team | 10 |
| 10 | Private App (Invitation only) | 1 |
| 11 | Not able to set an exercise program | 3 |
| 12 | Companion app | 5 |
| 13 | No demo available, expensive subscription | 1 |
| | **TOTAL** | 137 |

| App Name | Included (Y/N) | Reason for Exclusion |
|---|---|---|
| 110 Fitness | Y | |
| Cleo - My MS App | Y | |
| Connected mHealth | Y | |
| Daily Dose PD | Y | |
| Get Steady- Balance Exercises | Y | |
| LusioMATE | Y | |
| My Exercise Program | Y | |
| MyTRCare - Stroke Exercises | Y | |
| PhysiApp | Y | |
| Physio Ed. | Y | |
| Physiotec (My Wibbi) | Y | |
| PhysioTherapy Exercises (PTX) | Y | |
| Physiotools Trainer | Y | |
| Rehab Guru Client | Y | |
| Rephysio | Y | |
| Swiss Parkinson | Y | |
| Track Rehab (Rehab My Patient) | Y | |
| Yoga Vista App | Y | |
| AbbyCare | N | 1 |
| Aceso AI | N | 1 |
| Alliance care | N | 1 |
| Amp Recover | N | 1 |
| Barboza Method InStudio | N | 1 |
| Bauerfeind Training App | N | 1 |
| Body Engineering | N | 1 |
| Caroline Sweats | N | 1 |
| ComplexCore | N | 1 |
| CoreEX | N | 1 |
| Daily Feel Goods | N | 1 |

| App Name | Included (Y/N) | Reason for Exclusion |
|---|---|---|
| Embodia | N | 1 |
| Engage Health | N | 1 |
| Exercise Pro Live Engage | N | 1 |
| Full Body Workouts For Seniors | N | 1 |
| Guided Physio | N | 1 |
| Heal My Injury | N | 1 |
| HEFORA | N | 1 |
| iDIERS | N | 1 |
| Jen | N | 1 |
| Kinetisense PT Genie | N | 1 |
| Limber Health - Australia | N | 1 |
| Limber Health for Patients | N | 1 |
| Lu Strength & Rehab | N | 1 |
| My WebExercises | N | 1 |
| myHealthTrack | N | 1 |
| MyHEP PT Genie | N | 1 |
| Neuro Train | N | 1 |
| Neuromuscular Workout | N | 1 |
| NM Rehab Home Exercises | N | 1 |
| NOMS 360 | N | 1 |
| On The Mend | N | 1 |
| PtEverywhere | N | 1 |
| PT-Genie | N | 1 |
| ptMantra | N | 1 |
| Radian | N | 1 |
| Recovery | N | 1 |
| recovr: rehab and recovery | N | 1 |
| Renegade Rehabilitation | N | 1 |
| RunSmart | N | 1 |
| SimpleTherapy | N | 1 |
| SmartGym: Gym & Home Workouts | N | 1 |
| SmartPT Online | N | 1 |
| SpineTracker | N | 1 |
| The PT Initiative | N | 1 |
| The Steadman Clinic | N | 1 |
| TherEx Anywhere | N | 1 |
| TOA PT Genie | N | 1 |
| TrackActive Me: Virtual Physio | N | 1 |
| Traincor | N | 1 |
| True Sports Mobile | N | 1 |
| Universal Practice App | N | 1 |
| ViFive | N | 1 |
| Yogaia: Live Yoga Workouts | N | 1 |
| AC Health | N | 2 |
| Alfred, for ABI rehabilitation | N | 2 |
| Beats Medical Parkinson's App | N | 2 |
| CAVE Mobile App | N | 2 |

| App Name | Included (Y/N) | Reason for Exclusion |
|---|---|---|
| Digital MT | N | 2 |
| DIGITALBUDDY | N | 2 |
| Dive Brain Health | N | 2 |
| HealtheSteps | N | 2 |
| Mirror Box: CRPS & RSD, Stroke | N | 2 |
| MSAA—My MS Manager | N | 2 |
| neurosymptoms FND Guide | N | 2 |
| PhysioMate | N | 2 |
| PrepareMe | N | 2 |
| The MSing Link | N | 2 |
| Wheel With Me Fitness | N | 2 |
| Wysefit: Exercise for 50+ | N | 2 |
| Young Onset Parkinson's YOP-X | N | 2 |
| AllyCare | N | 3 |
| BlueJay Engage – Patient | N | 3 |
| Fiizio | N | 3 |
| Fusionetics | N | 3 |
| In Hand Health Patient App | N | 3 |
| My PTNOW | N | 3 |
| OneStep Physical Therapy | N | 3 |
| Physio 7 Training | N | 3 |
| Rehab Boost | N | 3 |
| RE-move: Patient | N | 3 |
| Wellpepper | N | 3 |
| X2U | N | 3 |
| YRMOVE | N | 3 |
| Ascenti Physio | N | 4 |
| ASPIRAction | N | 4 |
| Bespoke Health | N | 4 |
| Excelsior PT Patient App | N | 4 |
| Physio Clinic Exercises | N | 4 |
| Your SMHS Mobile Care | N | 4 |
| ATOMIC | N | 5 |
| ESPT | N | 5 |
| Extensor: The Physio App | N | 5 |
| GPEP Physiotherapy Exercises | N | 5 |
| Physio Pal | N | 5 |
| Sculpted | N | 5 |
| Sporti | N | 5 |
| ERVE Pro | N | 6 |
| Everyday workouts | N | 6 |
| Exercisify | N | 6 |
| HIT RUN APP | N | 6 |
| Kinisi | N | 6 |
| Performance PT App | N | 6 |
| Sport App Step | N | 6 |
| Stride Physical Therapy | N | 6 |

| App Name | Included (Y/N) | Reason for Exclusion |
|---|---|---|
| The Health Circle | N | 6 |
| Balance University | N | 7 |
| careplan.ai | N | 7 |
| Medicapp Cure | N | 7 |
| Neuro ProActive | N | 7 |
| Sweevy | N | 7 |
| ExorLive Assistant | N | 8 |
| physio.coach by Medbase | N | 8 |
| PTassistance | N | 8 |
| Rokoko Care | N | 8 |
| sophyapp | N | 8 |
| Virtual Training | N | 8 |
| BoBo Pro 2.0 | N | 9 |
| Motics (Physio) | N | 9 |
| PowerPlate Brain Bones Balance | N | 9 |
| Praep® Pods App | N | 9 |
| re.flex | N | 9 |
| Standout Balance | N | 9 |
| Standout Party | N | 9 |
| StepRite | N | 9 |
| Strive MedTech | N | 9 |
| Zibrio BalanceCoach | N | 9 |
| Kandu Health | N | 10 |
| MindMate | N | 11 |
| Rehabit: brain recovery habits | N | 11 |
| Simply Yoga - Fitness Trainer | N | 11 |
| Example Physio | N | 12 |
| Extensor | N | 12 |
| HelloFysio | N | 12 |
| Physitrack | N | 12 |
| Rehab Guru Pro | N | 12 |
| Health in Motion | N | 13 |
